# Does pharmaceutical advertising affect journal publication about dietary supplements?

**DOI:** 10.1186/1472-6882-8-11

**Published:** 2008-04-09

**Authors:** Kathi J Kemper, Kaylene L Hood

**Affiliations:** 1 Department of Pediatrics and the Program for Complementary and Integrative Medicine, Wake Forest University School of Medicine, Winston-Salem, NC, USA; 2 University of Florida, Department of Nutrition, Gainesville, FL, USA

## Abstract

**Background:**

Advertising affects consumer and prescriber behaviors. The relationship between pharmaceutical advertising and journals' publication of articles regarding dietary supplements (DS) is unknown.

**Methods:**

We reviewed one year of the issues of 11 major medical journals for advertising and content about DS. Advertising was categorized as pharmaceutical versus other. Articles about DS were included if they discussed vitamins, minerals, herbs or similar products. Articles were classified as major (e.g., clinical trials, cohort studies, editorials and reviews) or other (e.g., case reports, letters, news, and others). Articles' conclusions regarding safety and effectiveness were coded as negative (unsafe or ineffective) or other (safe, effective, unstated, unclear or mixed).

**Results:**

Journals' total pages per issue ranged from 56 to 217 while advertising pages ranged from 4 to 88; pharmaceutical advertisements (pharmads) accounted for 1.5% to 76% of ad pages. Journals with the most pharmads published significantly fewer major articles about DS per issue than journals with the fewest pharmads (P < 0.01). Journals with the most pharmads published no clinical trials or cohort studies about DS. The percentage of major articles concluding that DS were unsafe was 4% in journals with fewest and 67% among those with the most pharmads (P = 0.02). The percentage of articles concluding that DS were ineffective was 50% higher among journals with more than among those with fewer pharmads (P = 0.4).

**Conclusion:**

These data are consistent with the hypothesis that increased pharmaceutical advertising is associated with publishing fewer articles about DS and publishing more articles with conclusions that DS are unsafe. Additional research is needed to test alternative hypotheses for these findings in a larger sample of more diverse journals.

## Background

Interest in vitamins, minerals, herbs and other dietary supplements (DS) has grown markedly in the US since the early 1990's [1-10]. DS are defined by the US Food and Drug Administration as "vitamins, minerals, herbs or other botanicals, amino acids, and substances such as enzymes, organ tissues, glandulars, and metabolites." DS are commonly used [2,3,11,12]. In certain cases, DS are medically recommended (e.g., folic acid for women of child-bearing age [13]). However, for most supplements there are few official recommendations, and patients often use DS without informing their physician [2,14,15]. Physicians have been urged to discuss with their patients the effectiveness and safety of DS, particularly when combining use of DS with prescription medications [16-21].

In response to citizen demand and the high prevalence of DS use, the US National Institutes of Health have funded research to evaluate the safety and effectiveness of DS [22]. An increasing number of research papers about DS have been published recently, and a number of journals specifically devoted to CAM research and education have appeared. However, most primary care clinicians subscribe to established and highly visible general medical journals; articles in these journals are frequently cited in professional practice guidelines and by the media to guide the general public. Many medical journals are supported in part by advertising revenues; however, we are unaware of research evaluating the relationship between pharmaceutical advertising and publication about natural health products, which may in some cases be viewed as competitive with pharmaceutical products.

In general, the aim of advertising is to increase demand for and purchase advertised products. Direct to consumer pharmaceutical advertising affects consumer behavior [23,24] ; advertising in medical journals and other marketing aimed at clinicians affects clinician prescribing behavior [25,26]. Pharmaceutical funding of research also appears to influence authors' interpretation of results (conclusions) about the effectiveness of these products [27]. It is possible that advertising funds may influence journals and their paid staff [25]. The potential conflicts of interest between advertisers, editors and authors have resulted in the emergence of guidelines and rules about disclosure of potential conflicts of interest, particularly for authors writing about the effectiveness and safety of specific products [28-34].

Because of the importance of established medical journals in informing and influencing professional and public behavior, the question of unintended bias favoring advertisers in making editorial decisions is of great public health as well as business interest. The purpose of this pilot study was to begin to explore the relationship between pharmaceutical advertising and the publication of articles about DS. We expected that journals with more pharmaceutical advertising would publish fewer articles about DS because DS may at times be viewed as competitors to pharmaceutical products; furthermore, we expected that those with more pharmaceutical advertisements (pharmads) would publish more articles suggesting that DS were unsafe or ineffective than journals with fewer pharmads. As a secondary question, we assessed publications about DS in three journals devoted to complementary and alternative medicine (CAM); we expected CAM journals would have fewer pharmads and more DS articles; finally, we expected that fewer of the articles in CAM journals would be unfavorable about DS than articles in the general medical and primary care journals.

## Methods

### Data Sources

We selected 11 major medical journals published in English between June 30, 2006 and June 30, 2007. Journals were selected if they were devoted to general medicine *(American Family Physician, British Medical Journal, Canadian Medical Association Journal*, *Journal of the American Medical Association*, and *the New England Journal of Medicine*; internal medicine (*Annals of Internal Medicine *and *Archives of Internal Medicine*, or pediatrics (*Pediatrics, Archives of Pediatrics and Adolescent Medicine*, and *Pediatric Research*). For each journal, all issues available in the Coy C. Carpenter Library at Wake Forest University School of Medicine (WFUSM) for the study time period were reviewed. We used the American library version of these journals and did not compare different versions that may have been published for various groups of subscribers.

The WFUSM library did not subscribe to hard copies of any CAM journals. For the secondary question about CAM journals, we reviewed a convenience sample of three journals with issues published in the same time period as for the other journals to which the WFUSM Program for Holistic and Integrative Medicine at WFUSM had a subscription: *Journal of Alternative and Complementary Therapies*, *Alternative Therapies in Health and Medicine*, and *Explore*.

### Reviews

All reviews were carried out between May 21 and July 17, 2007. Journals were reviewed by two pre-medical students using standard rating forms created for the study. Reviewers were trained; inter-rater agreement was confirmed through independent review of journals. Reviewers examined each page of every journal reviewed. Pages were selected for the study if they included a) any kind of advertising or b) any report regarding a DS.

Advertising included pharmaceutical (including prescription and over-the-counter medications and ads for drug-eluting stents); classified; self-promoting (e.g., advertisements for the journal itself, its parent organization or an affiliated publication or meeting); for-profit products (e.g., foods, automotive, office equipment), non-profit (e.g., for schools, foundations, and associations such as the American Heart Association), and other (e.g. US military and other). For analytic purposes, these were collapsed into two categories: pharmaceutical versus other. Pages that contained advertising were counted as full, half (0.5) or quarter (0.25) ad pages for each type of ad.

Reports about DS were included if they were found in original manuscripts, reviews, editorials, abstracts, news, meeting notes, patient information, or letters to the editor. These materials are referred to for purposes of this paper as journal "articles" about DS. Indices and tables of contents were not included in the review. DS were defined as per the US FDA definition cited above. We excluded articles about tobacco, marijuana, alcohol products and about prescription medications derived from plants. We did not include articles about specific diets such as high fiber, low fat, low glycemic index, Mediterranean, Atkins, or specific foods such as milk, but we did include tea and coffee because many of these articles focused on their caffeine or antioxidant content. For analytic purposes, articles about DS were divided into major articles (original research, editorials, reviews) and other (basic scientific mechanisms, case reports, letters, fillers, news, abstracts, meeting notes, and similar) types of articles. We divided articles this way because original articles, reviews, and editorials tend to be picked up by the media and cited in subsequent review articles; even though editorials and reviews do not often include new data, they often contribute to policy and guideline development.

For each article about DS, reviewers examined the authors' conclusions regarding safety and effectiveness. Conclusions about safety were coded as a) unsafe, b) safe, or c) unclear or mixed or d) not discussed. For example, a conclusion citing "no serious adverse effects" was coded as "safe"; a conclusion that excessive vitamin A caused liver disease would be coded as "unsafe"; some articles that focused solely on efficacy did not include a discussion of side effects. Similarly, conclusions about effectiveness were coded as a) ineffective, b) effective, or c) neutral, mixed or insufficient evidence or d) not discussed. Reviewers discussed cases in which there was initial disagreement about coding (<5% of cases); the two unresolved cases were referred to an attending physician for final decision about coding. Because we were particularly interested in negative publications, articles were categorized for analysis as "unsafe" versus "other" and as "ineffective" versus "other."

Data about each journal's impact factor was collected for 2006 on the journal websites.

Data were entered into a MS Access Database and transferred to MS EXCEL for descriptive analysis; Stata™ 8.1 software was used for statistical analysis. Comparisons between groups of journals with the most, middle and fewest articles per issue were made with Fisher's exact test due to the small sizes of some cells.

As an analysis of published materials, this study was considered exempt from human subjects review.

## Results

The number of issues reviewed for each of the 11 primary journals ranged from 12 to 52, with an average of 30 issues per journal (Table 1). The average number of pages per issue ranged from 56 to 217. The average number of all advertising pages (all-ads) ranged from 3.9 to 87.8 per issue. The average number of pharmaceutical ads (pharmads) varied from 0.15 to over 60 pages per issue and from 0.001 to 0.441 pharmads per average number of journal pages per issue. We arbitrarily grouped journals into those with less than ten pharmads in an average issue (the fewest pharmads group; two journals with an average impact factor 5.9); those with more than 40 pages of pharmaceutical ads per issue (the most pharmads group; two journals with an average impact factor 18.5), and those that had between 10–39 pages per issue (the middle ads group; seven journals with an average impact factor 6.9). Although we did not set out to review the number of pages devoted to DS advertising, reviewers noted that ads for DS such as Centrum^® ^and Caltrate^® ^were very rare among these 11 journals, and there were no ads for folate, individual vitamins or for non-vitamin/mineral DS such as glucosamine, ginkgo, or garlic in these 11 journals.

**Table 1:** Journal advertising – Total pages and Pharmaceutical Ads (Pharmads)

**Journal**	**No. of issues reviewed**	**Average No. of Pages per issue**	**Average No. of All Ad pages per issue**	**Average No. Pharmads per issue**	**Percent Pharmad pages per issue**	**No. of Pharmads per Pages in Avg. issue**
A	24	182.9	87.8	66.3	75.5	0.362
B	25	143.4	86.7	63.2	72.9	0.441
C	52	108.4	85.0	39.4	46.4	0.363
D	24	78.8	53.2	22.8	42.8	0.289
E	12	217.3	63.6	24.7	38.9	0.114
F	12	109	21.7	15.7	72.3	0.144
G	22	117.7	21.0	15.4	73.3	0.131
H	50	86.1	22.2	15.2	67.5	0.177
I	48	124.8	30.8	10.5	34.0	0.084
J	52	56.1	3.9	0.15	3.9	0.003
K	13	137.5	10.5	0.15	1.5	0.001

***OVERALL AVERAGE for major medical journals***	***30.4***	***123.8***	***43.04***	***23.5***	***48.1***	***0.192***

***CAM journals***				***Pharmads (DS ads) per issue***	***Percent pharmads (DS ads)***	

L	6	79.2	8	0 (1.8)	0 (23)	0
M	7	103.7	5.7	0 (0)	0 (0)	0
N	9	104.1	24	0 (12.3)	0 (52)	0

The number of articles of *any *type about any DS in each journal ranged from four to 61 (Table 2). The journals with the most pharmads published fewer *major *articles about DS per issue than journals with a middle number of ads (0.08 versus 0.21 per major articles per issue, P < 0.05); and journals with the middle number of pharmads published fewer major DS articles per issue than journals with the fewest pharmads (0.21 versus 0.43, P < 0.05), consistent with a dose-effect response. There was no apparent relationship between journals' impact factor and either their number of ads or their number of publications about DS. None of the 35 articles published about DS in the two journals with the most pharmads were clinical trials or observational cohort studies.

**Table 2:** Articles about Dietary Supplements (DS)

**Characteristics**	**Number of issues reviewed**	**Number of ANY articles about DS**	***Clinical Trials or Cohort Studies***	***Editorials***	***Reviews***	TOTAL MAJOR ARTICLES (PER ISSUE)*
*Most Pharmads (2 journals; average impact factor 6.9) TOTALS*	49	35	0	2	2	4
*Number per issue*		**0.71**	**0**	**0.02**	**0.02**	(0.08)*
*Middle Pharmads (7 journals; average impact factor 18.5) Totals*	220	142	30	15	2	47
*Number per issue*		**0.65**	**0.14**	**0.07**	**0.01**	(0.21)*
*Fewest Pharmads (2 journals; average impact factor 5.9) Totals*	65	77	14	11	3	28
*Number per issue*		**1.2**	**0.22**	**0.17**	**0.05**	(0.43)*
						
***CAM JOURNALS***	22	88	11	2	6	19
*Number per issue*		**4**	**0.5**	**0.09**	**0.27**	(0.86)*

*Overall differences in rates of publishing *major *articles about DS are statistically significant with P < 0.001. The rate for those with the most pharmads (0.08) is significantly lower than those with middle pharmads (0.21, P < 0.05). The rate for those with the middle pharmads (0.21) is significantly lower than those with the fewest pharmads (0.43, P < 0.05). The rate for the CAM journals (0.86) is significantly higher than that for the general medical journals with the fewest pharmads (<0.01)

The DS that were covered the most often in these medical journals were (Table 3): folate and other B vitamins; calcium and vitamin D; iron; essential fatty acids such as omega three fatty acids; and caffeinated beverages such as coffee and green tea. There were fewer articles about probiotics, glucosamine, individual herbs (garlic, ginkgo, ginseng, St. Johns wort, lavender, tea tree oil) or combination herbal products.

**Table 3:** Dietary Supplements Most Often Covered in Major Medical Journals

**Supplement**	**Number of Articles**
Folate and other B vitamins	37
Coffee, green tea, caffeine	20
Calcium and/or Vitamin D	18
Iron	11
Omega-3 fatty acids	9
Multivitamins or multiple vitamin/nutrient mixtures	6
Lavender/tea tree	6
Vitamin A	5
Zinc	5

Other DS included probiotics, olive oil, antioxidants, garlic, Vitamins C, E, and K chondroitin, and black cohosh

Safety concerns were raised less often in clinical trials or cohort studies than in other articles. Specifically, concerns about unsafe DS were cited in 1/44 (2%) clinical trials or cohort studies versus 3/34 (10%) of editorials or reviews, and 20/177 (11%) of other kinds of articles. Journals with the most pharmads were significantly more likely to publish major articles concluding that DS were *unsafe *than journals with middle or fewest pharmads (67% versus 7% and 4%, respectively, P < 0.005 Table 4).

**Table 4:** Articles concluding DS are *Unsafe *or *Ineffective*

**Characteristics**	**N *Unsafe*/All DS articles (%)**	**Among Clinical or Cohort Trials**	**Among Editorials**	**Among Reviews**	***N Unsafe/TOTAL MAJOR ARTICLES***
***UNSAFE***					

*Most Pharmads*	5/35 (14)	0/0 (0)	1/1 (100)	1/2 (50)	2/3 (67)*
*Middle Pharmads*	16/142 (11)	1/30 (3)	0/15 (0)	0/2 (0)	**1/47 (7)**
*Fewest Pharmads*	3/77 (4)	0/14 (0)	1/11 (9)	0/3 (0)	**1/27 (4)**
					
*CAM Journals*	1/88 (1)	0	0	0	**0/19 (0)**

***INEFFECTIVE***	**N *Ineffective*/All DS articles (%)**				***N Ineffective/TOTAL MAJOR ARTICLES***

*Most Pharmads*	12/35 (34)	0/0	1/2 (50)	0/2 (0)	**1/4 (25)**
*Middle Pharmads*	35/142 (25)	8/31 (26)	5/15 (33)	1/2 (50)	**14/47 (30)**
*Fewest Pharmads*	21/77 (27)	2/14 (14)	3/11 (27)	0/3 (0)	**5/27 (19)**
					
*CAM Journals*	11/88 (13)	1/11 (9)	0/2 (0)	1/6 (17)	**2/19 (11)**

* General medical journals with the most pharmads published a significantly higher percentage of major articles concluding that DS were unsafe than the journals with middle or fewest pharmads or the CAM journals (P < 0.005).

The differences in rates of articles concluding that DS were ineffective among the different pharmad groups were not statistically significant.

In the 11 primary journals, 68/254 (26%) articles about DS concluded they were *ineffective; *only 10 of these articles were clinical trials or cohort studies. Conclusions about ineffective DS were less common in journals with fewest pharmads than in journals with more pharmads. For example, half (50%) of the editorials from journals with the most pharmads concluded that DS were ineffective, compared with 33% of editorials from journals with middle amounts of pharmaceutical advertising and 27% of editorials from the journals with the least pharmaceutical advertising (Table 4); these differences were not statistically significant.

For the secondary question about the three CAM journals, the number of advertisements ranged from 5.7 to 24 pages per issue (Table 1). There were no advertisements for prescription pharmaceuticals in any of the CAM journals. However, there were ads for DS; the number of pages of ads for DS ranged from 0 to 12.3 with an overall average of 5.5 ads for DS per issue. The CAM journals carried significantly more articles about DS than any group of the primary journals (P < 0.01). The CAM journals published over twice as many clinical trials or cohort studies as the journals with the fewest pharmads (0.5 versus 0.22 per issue); the number of *major *articles per issue was significantly higher among CAM journals than among the general medical journals with the fewest ads (0.86 versus 0.43 P < 0.05). Although CAM journals published more original science about DS, the proportion of major articles concluding that DS were unsafe or ineffective was not significantly different from general journals with middle or fewest pharmads (Table 4).

## Discussion

This is the first study to examine the relationship between advertising in medical journals and the publication of articles in those journals about DS. The results are consistent with the hypothesis that pharmaceutical advertising biases journals against non-drug therapies. Journals with the most pharmads per issue published significantly fewer major articles about DS than journals with fewer pharmads. For example, the two journals with the most pharmads published no clinical trials or cohort studies about DS. Furthermore, journals with the most pharmads were more likely to publish articles concluding that DS were unsafe than journals with fewer pharmads. CAM journals had no pharmaceutical ads, and they published significantly more original science articles about DS; however, they were not significantly less likely to publish major articles suggesting that DS were unsafe or ineffective than general medical journals with middle or few pharmads.

These results are generally consistent with a substantial body of research suggesting that advertising influences behavior. Advertising influences consumer behavior [23,24]. Advertising, particularly in the forms of gifts and samples, also influences physician prescribing behavior [26,35,36]. This study extends those findings, and it supports growing efforts to minimize potential conflicts of interest or at least make them more transparent, if eliminating them entirely is not feasible [28,37,38].

It is possible that there might have been greater apparent impact on publication decisions if the analysis had been restricted to DS that are typically used to replace pharmaceutical products. The great majority of the articles about DS reviewed here were about caffeinated beverages and vitamins or other essential nutrients (minerals and essential fatty acids) which are not competitors of prescription medications. For the most part, recent research suggests that the DS most often used, including multivitamins, calcium, iron and folate, are those often recommended by professionals or those known to be deficient in the diet [39-41]. Furthermore, many vitamins are manufactured by pharmaceutical companies, thus reducing these companies' potential bias against DS. For example, Centrum^® ^products and Caltrate^® ^are manufactured by Wyeth, the maker of Advil^®^, Alavert^®^, Effexor^® ^and Embrel^® ^among many other products. These facts would tend to favor the null hypothesis.

On the other hand, non-vitamin/mineral supplements, such as glucosamine, echinacea, garlic, ginseng, and ginkgo are also frequently used by the public, often concurrently with prescription medications [12,20], yet the primary 11 journals included in the survey published few research studies, editorials or reviews on these DS. Future research will need to focus on publication about DS that may be viewed as greater threats to the pharmaceutical industry (such as non-vitamin/mineral products that are used by the public to promote weight loss, improve sexual or athletic performance, relieve joint or back pain, or to improve memory, cardiovascular health or GI health). Such research will need a large sample size of many types of journals; furthermore, such research will also need to account for potential confounding by research funding by DS manufacturers.

This pilot study has several limitations. It included only a few of the many clinical medical journals available and used only the American library version of these journals, but it did focus on those with very high readership that are often quoted by professional organizations, the media and policymakers. Results from the smaller secondary analysis of the CAM journals would be enhanced by larger sample of more diverse CAM publications. The review did not specifically address the funding for different trials or evaluate the methodologic quality of the published trials. This study was not a survey of journal editors regarding the number of submissions of different types they receive or how they decide which articles to include. An alternative hypothesis that might explain these findings is that journals with a high number of pharmaceutical ads receive few submissions about dietary supplements; furthermore, it is possible that a large number of articles about dietary supplements are of poor quality and do not deserve publication. It is also possible that the observed associations are due to another underlying factor ; for example, one recent study reported that in high impact journals, European journals were approximately twice as likely as American journals to publish positive articles about complementary therapies [42]. In addition, this study used the number of issues per journal as the study denominator rather than the total number of articles per issue; future studies could use the number of articles per issue as the denominator to address the related question of whether journals with more advertising per issue also have fewer articles of all types and hence fewer articles about dietary supplements. Future studies on this topic to explore alternative hypotheses for our findings should include a larger number of journals and more diverse journals; additional studies might also survey journal editors to determine the number of articles submitted for consideration for publication and ask about the percentage of submissions related to dietary supplements.

## Conclusion

These findings support the hypothesis that in major medical journals, more pharmaceutical advertising is associated with publishing fewer articles about DS and having more negative conclusions about DS safety. While awaiting future definitive studies to confirm these findings in a larger, more diverse sample of journals and to explore alternative explanations, these data support current efforts to reduce conflicts of interest in medical publishing and to make any such conflicts more transparent. The impact of advertising on publications appears to be non-trivial; the ultimate impact of this bias on professional guidelines, health care, and health policy is a matter of great public concern and underscores the need for additional health services research on this topic.

## Competing interests

The author(s) declare that they have no competing interests.

## Authors' contributions

KK conceived of the project, designed the data collection form, reviewed data analysis, drafted and revised the manuscript

KH reviewed journals, entered data, assisted with data analysis and manuscript preparation

Both authors read and approved the final manuscript.

## Pre-publication history

The pre-publication history for this paper can be accessed here:


